# Dried Matrix Spots for the Determination of Opiates and Opioids: Methodological Advances and Applications

**DOI:** 10.3390/molecules30183695

**Published:** 2025-09-11

**Authors:** Luana M. Rosendo, Rita Gonçalves, Rodrigo Martins, Vitória Castro, Tiago Rosado, Mário Barroso, Eugenia Gallardo

**Affiliations:** 1 RISE-Health, Departamento de Ciências Médicas, Faculdade de Ciências da Saúde, Universidade da Beira Interior, 6200-506 Covilhã, Portugal; may.rosendo@ubi.pt (L.M.R.); rita.alexandra.paula.goncalves@ubi.pt (R.G.); rodrigo.coelho.martins@ubi.pt (R.M.); vitoria.castro@ubi.pt (V.C.); 2 Laboratório de Fármaco-Toxicologia, UBIMedical, Universidade da Beira Interior, 6200-000 Covilhã, Portugal; 3 Centro Académico Clínico das Beiras (CACB)-Grupo de Problemas Relacionados com Toxicofilias, 6200-000 Covilhã, Portugal; 4 Serviço de Química e Toxicologia Forenses, Instituto Nacional de Medicina Legal e Ciências Forenses-Delegação do Sul, 1169-201 Lisboa, Portugal; mbarroso@alphabiolabs.com; 5 AlphaBiolabs, 14 Webster Court, Carina Park, Warrington WA5 8WD, UK

**Keywords:** dried matrix spots, opioids, opiates, bioanalytical, drug monitoring

## Abstract

Dried matrix spot (DMS) techniques have gained increasing attention in bioanalytical and forensic toxicology for the detection of opiates and opioids, offering minimally invasive sampling, enhanced sample stability, and simplified storage and transport. This review provides a critical overview of recent methodological advances and applications of DMS across multiple biological matrices, including blood, plasma, urine, and oral fluid. Particular focus is given to sample preparation protocols, extraction strategies, analytical instrumentation, and method performance. Dried blood spots (DBS) remain the most established format; however, alternative matrices such as dried plasma, urine, and saliva spots (DPS, DUS, DSS) are expanding the scope of DMS, particularly in decentralised and point-of-care contexts. Despite clear advantages, such as reduced biohazard risk and compatibility with high-throughput workflows, several limitations persist, including low sample volumes, matrix-specific recovery issues, and lack of standardised procedures. Future efforts should aim to optimise paper substrates, improve solvent–matrix compatibility, and integrate DMS workflows with automated or miniaturised mass spectrometry platforms. Overall, DMS techniques represent a versatile and evolving analytical platform with strong potential for reliable opioid monitoring in both clinical and forensic settings.

## 1. Introduction

The widespread use of opiates and opioids—ranging from therapeutic agents such as morphine, codeine, and fentanyl to illicit substances like heroin—has created major public health challenges worldwide [1,2]. The opioid epidemic, particularly evident in North America but increasingly global in scope, highlights the urgent need for effective tools for drug monitoring, compliance assessment, and toxicological surveillance [3]. Analytical methodologies for detecting these compounds must strike a balance between sensitivity, specificity, sample integrity, and logistical feasibility [4,5].

Traditional biological matrices used in drug testing—such as blood, plasma, urine, and oral fluid—provide well-established platforms for analysis but also present several practical limitations. These include the need for trained personnel for sample collection, potential biohazard risks during transport and handling, reliance on cold-chain storage to ensure analyte stability, and challenges associated with field or home-based sampling. Such drawbacks are particularly problematic in decentralised studies, longitudinal follow-ups, paediatric or geriatric populations, and remote geographic areas [3,6,7,8].

To overcome these limitations, dried matrix spot (DMS) techniques have emerged as a robust alternative for bioanalytical sampling [9]. The fundamental principle involves depositing small volumes of biological fluids onto porous supports, typically cellulose-based filter papers, followed by ambient drying and subsequent extraction for analytical purposes. By converting liquid biological samples into a solid, dried format, DMS offers significant advantages in terms of sample stability, ease of storage and transport, minimal biohazard risk, and reduced sample volume requirements [10,11,12].

The origin of DMS dates back to the 1960s, with the introduction of dried blood spots (DBS) for neonatal screening by Robert Guthrie [6]. Since then, the technology has undergone continuous refinement and expansion, extending its applicability to other matrices such as urine (DUS), plasma (DPS), and oral fluid (DSS). In recent years, there has been growing interest in applying DMS strategies to the detection of opiates and opioids, particularly given their chemical diversity, variable stability, and increasing relevance in both forensic and clinical contexts [9,11].

Despite these advances, the application of DMS to opioid analysis remains an evolving field, with critical knowledge gaps regarding analyte stability, matrix effects, method standardisation, and regulatory validation. Moreover, synthetic opioids and emerging new psychoactive substances (NPS) pose unique analytical challenges that demand further methodological innovation [5,10,13,14].

This review provides an up-to-date overview of methodological developments in DMS techniques for the determination of opioids, while also addressing current analytical limitations and proposing future directions to enhance their robustness and applicability. By integrating historical background with technical evaluation, it aims to offer a critical and original perspective that goes beyond conventional literature reviews.

## 2. Biological Matrices in DMS Applications

DMS techniques have been increasingly applied to the determination of opiates and opioids, reflecting both the analytical challenges posed by these compounds and the clinical and forensic contexts in which they are monitored [1,2,3]. The choice of matrix is critical, as it influences analyte stability, sample preparation requirements, and interpretative value.

Blood is considered the reference matrix for opioid monitoring, as it reflects the pharmacologically active fraction of the drug at the time of sampling [9,10]. Morphine, codeine, methadone, and synthetic opioids such as fentanyl analogues are commonly quantified in blood to assess acute intoxication or therapeutic compliance [10,15]. It is also frequently employed to validate alternative matrices, given its ability to correlate analyte concentrations with clinical effects [10,15]. However, blood collection is invasive, requires trained personnel, and poses logistical difficulties related to refrigeration and transport [10,16]. Matrix effects, such as ion suppression or protein binding, must also be controlled during analysis [17]. Nonetheless, blood-based DMS methods, particularly DBS, provide a more stable and logistically advantageous alternative to liquid samples, offering benefits such as improved stability of opioids (including labile compounds like heroin metabolites) and a reduced biohazard risk [9,10].

Plasma and serum are frequently used to evaluate systemic exposure to opioids, enabling concentration–effect correlations that are essential in clinical toxicology and therapeutic drug monitoring (TDM) [9,15]. However, these matrices also require invasive venepuncture, and the presence of proteins can lead to drug binding that complicates interpretation [9,10]. Protein binding and matrix effects remain important considerations, particularly for lipophilic opioids such as fentanyl, which require careful optimisation of extraction conditions [5].

The application of dried plasma spots (DPS) has improved sample handling and stability while maintaining analytical robustness, offering an attractive alternative for specific clinical and research scenarios [10,18]. For instance, DPS have been successfully applied to the monitoring of oxycodone and oxymorphone, providing simplified storage and transport without compromising analytical robustness [9].

Urine is widely used in routine toxicological assessments due to its non-invasive collection, larger available volume, and high concentrations of excreted metabolites [9,10]. It offers an extended detection window for many substances and is commonly employed to monitor drug use over time [9]. As opioids and many other drugs are often excreted as glucuronide conjugates, enzymatic hydrolysis is typically required to improve sensitivity and accuracy [9,15]. Codeine and morphine are frequently targeted in dried urine spots (DUS) applications. However, urinary concentrations do not directly reflect pharmacological impairment and are subject to variability due to hydration status, renal function, and metabolic rate [9,10]. Risks of adulteration and analyte degradation during transport and storage also exist. These limitations can be mitigated by using DUS, which offer enhanced stability and simplify transport and storage logistics, particularly in decentralised or field-based settings [10].

Oral fluid (OF), often referred to as saliva in the context of DMS techniques, is another matrix increasingly used in analytical applications. Although the term “dried saliva spot” (DSS) is commonly used, it refers specifically to oral fluid collection. OF reflects the unbound, pharmacologically active fraction of drugs in circulation due to the high vascularisation of the salivary glands [10,12]. Lipophilic and low-molecular-weight compounds, such as opioids, can diffuse into OF, and weakly basic drugs may undergo ion trapping due to its acidic pH, thereby enhancing detectability [10]. OF sampling is simple, painless, and can be performed without medical personnel, making it ideal for roadside or workplace testing [10]. However, small sample volume, interindividual variability, and contamination risk remain limiting factors. The development of DSS techniques has addressed some of these issues by improving sample stability and facilitating both transport and long-term storage [12,19].

In addition to these commonly studied matrices, other biological fluids have also been explored in the context of DMS. Cerebrospinal fluid (CSF), although rarely used, offers valuable insights into neuropharmacological research and cases involving central nervous system exposure to drugs. However, its highly invasive collection limits its use to specialised clinical investigations [10,15]. Tears have also been proposed for therapeutic drug monitoring and ocular pharmacokinetics, benefiting from easy and non-invasive collection, though their application in DMS remains largely experimental [12]. In forensic and *postmortem* contexts, vitreous humour and pericardial fluid have also been used for the determination of *R*- and *S*-methadone, as well as their metabolite *R*-/*S*-EDDP, with dried spot approaches demonstrating improved analyte stability compared with whole blood [9,18]. These matrices, when considered collectively, highlight the adaptability of DMS methodologies; however, their use remains limited to highly specific scenarios and requires further validation before broader implementation in clinical or forensic settings.

Overall, the selection of an appropriate matrix for DMS depends on the analytical objective, drug properties, collection feasibility, and logistical constraints. Each biological fluid presents unique characteristics that may favour its application under specific conditions. The increasing adoption of DMS techniques across diverse matrices reflects their growing relevance in modern toxicological, pharmacological, and diagnostic settings.

## 3. Dried Matrix Spot Techniques

DMS techniques have progressively expanded beyond blood-based formats to encompass a wide range of biological matrices, including urine, plasma, OF, tears, breast milk, CSF, and even synovial fluid. Each of these matrices presents distinct physicochemical characteristics, such as viscosity, protein content, and analyte distribution, which must be considered during method development [7,20,21]. While DBS, DUS, and DPS are the most commonly reported formats, emerging variants such as DSS, dried breast milk spots (DBMS), and dried cerebrospinal fluid spots (DCSF) reflect the expanding versatility of this sampling strategy. However, the successful application of DMS across such diverse matrices requires a detailed understanding and careful optimisation of several technical parameters. This matrix versatility has demonstrated considerable analytical advantages across multiple fields, particularly in forensic toxicology, drug and biomarker monitoring, and clinical research applications [10,12,22,23,24].

Among these, DBS remains the most established approach, typically collected via capillary puncture, such as a finger prick. DBS has been extensively applied in clinical and pharmacokinetic studies due to its practicality and the substantial validation literature supporting its use [7,8,11,25]. However, its quantitative accuracy can be significantly affected by the haematocrit (Hct) effect, which alters blood viscosity and spreading behaviour on filter paper, leading to non-uniform analyte distribution across the spot [20,21]. Moreover, spot homogeneity remains a critical factor for reproducibility, particularly when standardised punch areas are used for analyte extraction [10,20,26].

DUS provides a broader detection window for opioid metabolites, thus allowing retrospective monitoring of drug intake over extended periods. This format is particularly valuable in drug abuse screening and forensic toxicology [24,26]. Nonetheless, it presents analytical challenges such as potential analyte redistribution during the drying process and variable dilution due to individual hydration status, both of which may affect quantitative consistency [27].

In contrast, DPS are prepared by separating plasma from whole blood before spotting. This approach reduces matrix complexity and eliminates Hct-related variability, thereby enhancing analytical precision. However, it requires additional processing steps, limiting its use in decentralised settings [12,20,21,24].

More recently, DSS has emerged as a promising alternative for non-invasive drug monitoring. OF reflects recent drug use and has the advantage of being easy to collect without the need for trained personnel [12,19]. Nevertheless, salivary concentrations of opioids are typically lower than those found in blood or urine. These concentrations are influenced by pH, flow rate, and the physicochemical properties of the drug, which can complicate method development and validation. The heterogeneity and variability inherent to each matrix necessitate customised protocols and validation for each DMS format, particularly when targeting compounds with diverse pharmacokinetic profiles such as opiates and opioids [12,19].

Comparative studies further illustrate these challenges. Almeida et al. [28] reported that in DSS, 6-MAM degraded almost completely within a few days at room temperature, whereas morphine and codeine exhibited losses of 20–40% over 30 days, highlighting the impact of pH and storage on analyte stability. Ribeiro et al. [29] further showed that recoveries in DSS ranged between 54 and 74%, reflecting the strong influence of saliva-specific factors such as pH and secretion variability on analytical performance. These findings confirm that while DSS offers clear advantages in non-invasive sampling, rigorous validation against liquid oral fluid is required before broader forensic or clinical application.

**Figure 1:**
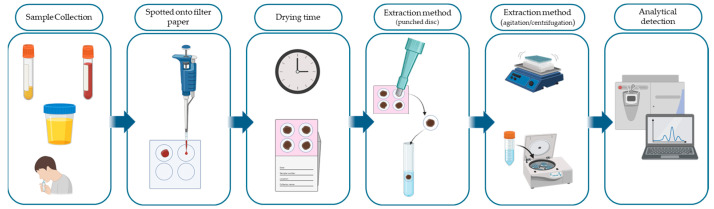
General workflow of dried matrix spot (DMS) sample preparation and analysis.

Furthermore, although infrequently, other alternative matrices, such as cerebrospinal fluid, tears, vitreous humour, or pericardial fluid, have also been investigated. These matrices are emphasised as potential but extremely context-specific solutions that still need significant evaluation before wider implementation, as their viability and limits have been covered in Section 2. The overall workflow for DMS analysis involves multiple sequential steps that must be standardised to ensure analytical robustness. These steps are summarised in Figure 1, which outlines the typical sequence from sample collection to instrumental detection.

The process begins with a sample application, in which a defined volume, usually between 10 and 50 µL, is deposited onto pre-marked zones of filter paper using calibrated micropipettes or by non-volumetric application methods that rely on subsequent fixed-diameter punching [10,12,24]. Homogeneous sample deposition is essential to minimise intra-spot variability and ensure reproducible analyte recovery. The drying phase is typically conducted under ambient laboratory conditions (20–25 °C) for a minimum of two to four hours. This step should take place in a clean, dust-free environment, with passive or active airflow to accelerate drying and prevent microbial growth or chemical degradation [10,12,24].

Once dried, samples are stored in sealed containers, often in zip-lock plastic bags containing desiccant sachets to control humidity. Depending on analyte stability, storage may occur at room temperature, 4 °C, or −20 °C. For opioids sensitive to oxidative degradation or photolysis (e.g., 6-MAM, morphine, codeine), protection from light is also recommended. A major advantage of the DMS format is its compatibility with ambient transport conditions, eliminating the need for cold-chain logistics. This feature greatly facilitates sample shipment in multicentric studies, remote locations, and home-based collection settings [12].

To ensure analytical reliability, several critical parameters must be optimised during method development. The first consideration is the volume and homogeneity of the applied sample, which directly influence spot morphology, analyte distribution, and extraction efficiency [21,24]. Deviations in spot volume can lead to variable matrix-to-analyte ratios, impairing quantification. For high-throughput workflows, automation of the spotting process is strongly recommended.

Equally important is the choice of filter paper. Different brands and compositions—such as Whatman 903 or FTA DMPK cards—vary in porosity, absorption capacity, potential for analyte binding, and recovery efficiency. These properties can significantly affect analyte retention, elution, and stability, and must therefore be matched to the physicochemical characteristics of the target compound (e.g., polarity, lipophilicity, ionisation behaviour) [12,24].

In DBS, the haematocrit effect remains one of the most discussed limitations. Hct values influence the viscosity of blood and its diffusion on paper. Elevated Hct levels increase blood viscosity, resulting in smaller and denser spots, concentrating the analyte in the centre, whereas low Hct levels lead to wider dispersion and potential underestimation [20,24,25]. This variability introduces challenges in quantitative accuracy, especially when fixed-diameter punches are used for analysis. Strategies to mitigate this include volumetric absorptive microsampling (VAMS), which ensures fixed-volume collection independent of spreading, or the application of correction algorithms based on visual or spectrometric spot assessment [30,31].

A validated model developed by Alsous et al. [20] demonstrated that haematocrit values between 25 and 55% and blood volumes of 7.5–30 µL significantly altered the surface area of DBS, with higher Hct reducing spot size and leading to artificially elevated analyte concentrations. In opioid analysis, this heterogeneity may result in underestimation or overestimation of drug exposure, with direct implications for therapeutic drug monitoring and forensic interpretation. Protti et al. [9] compared blood, plasma, and urine analysed in both dried and liquid formats, reporting that oxycodone, noroxycodone, and oxymorphone were stable for up to 30 days in DPS and DUS, while signal losses of up to 20% occurred in dried urine spots during longer storage, potentially leading to underestimation of chronic use or false negatives in forensic investigations. These findings illustrate how technical limitations in DMS can directly impact clinical and forensic decision-making, emphasising the need for rigorous validation. The position and size of the punched area used for extraction also affect accuracy and precision. Since analyte distribution is rarely uniform across the spot, central punches are typically used to reduce edge-related variability. In some protocols, full-spot extraction is employed to avoid this issue altogether, although this precludes sample reusability [7,20].

Extraction conditions must be meticulously optimised. Parameters such as solvent type (e.g., methanol, acetonitrile, aqueous buffers), pH, extraction volume, agitation time, and temperature all influence recovery and matrix cleanliness [12,19,24]. For opioid analysis, acidified organic solvents often enhance analyte solubility and stability, but these conditions must be balanced against the risk of degradation or co-extraction of matrix interferences. Optimisation should also consider extraction efficiency, reproducibility, and compatibility with detection platforms, particularly when using liquid chromatography-tandem mass spectrometry (LC–MS/MS) or gas chromatography-tandem mass spectrometry (GC–MS/MS) [4,10,13,32].

Finally, the stability of each analyte under various storage and environmental conditions must be validated individually. Opioids, especially synthetic analogues such as fentanyl derivatives or nitazenes, can be chemically unstable and prone to degradation via hydrolysis, oxidation, or photolysis [13,33]. Vitrano et al. [13] reported that nitazene derivatives degraded almost completely (>80% loss) within 30 days at room temperature (1 ng/mL), while storage at 4 °C only partially improved stability. Truver and Swortwood-Gates [33] demonstrated that several synthetic opioids showed losses greater than 30% under non-refrigerated conditions. Palmquist and Swortwood [34] further observed substantial long-term instability among 13 fentanyl analogues in blood: under refrigerated conditions, they remained stable for up to nine months (81–112% recovery), whereas at elevated temperatures, most of the analytes were stable for one week, confirming that storage temperature is a decisive factor. Complementary evidence from Almeida et al. [28] showed that 6-MAM in DSS degraded almost completely within a few days at room temperature, with significant signal losses (>20%) after 24 h, whereas morphine and codeine displayed more gradual decreases over 30 days (20–40% loss). Light exposure accelerated degradation, while storage at −20 °C and the addition of sodium fluoride markedly improved stability. Stability studies should include short-term exposure, long-term storage, freeze–thaw cycles, and transportation simulation [33,34].

Additionally, carry-over and contamination are key concerns in high-throughput laboratories. Cleaning of punch tools or the use of disposable devices is essential to avoid false positives or cross-contamination, particularly when operating at low detection limits [8].

**Figure 2:**
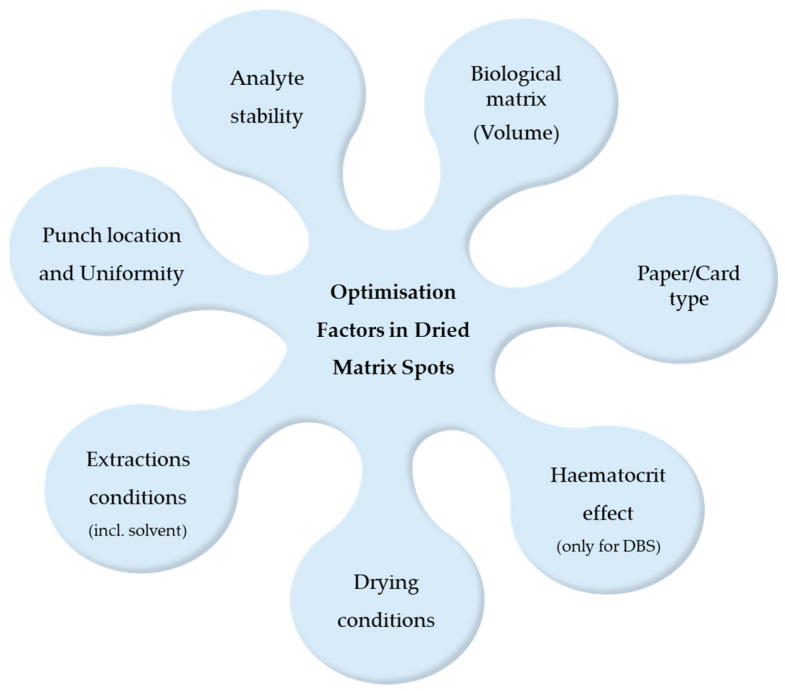
Summary of key optimisation factors in DMS techniques.

Through the careful optimisation of these technical variables, DMS techniques can provide reliable, cost-effective, and minimally invasive alternatives for the quantification of opiates and opioids in a variety of analytical contexts [3,9,29]. A visual summary of the main optimisation factors in DMS method development is presented in Figure 2. While several of these parameters (such as paper type, sample volume, and drying and extraction conditions) are further detailed in the study summaries presented in Section 4, other aspects are more appropriately illustrated in a schematic format to provide a conceptual overview. In this way, the figure complements the tabulated information by offering both a conceptual and a practical perspective.

## 4. Analytical Methods and Applications

All articles reviewed in this section were identified through systematic searches conducted in PubMed and Scopus, covering the period from 2015 to 2025. The search strategy included the following keywords: opioids, dried blood spots, human, dried urine spots, dried plasma spots, dried matrix spots, and dried saliva spots. Only original research articles applying DMS to the determination of opiates and opioids in biological matrices were included. Review articles, studies based solely on synthetic or artificial matrices, and works not employing DMS techniques were excluded. DMS methodologies have gained substantial relevance in bioanalytical workflows for opioid detection due to their minimal sample requirements, enhanced analyte stability, and compatibility with decentralised collection strategies. This section summarises and critically evaluates the analytical performance of DMS-based methods for opioids across different biological matrices. To highlight matrix-specific challenges and advantages, the discussion is structured into two parts: (1) DBS, as the most established format; and (2) alternative matrices such as urine, plasma, and OF.

### 4.1. Overview of Methods Based on Dried Blood Spots

DBS remains the most widely implemented DMS format due to its well-established role in clinical toxicology and forensic applications. The studies summarised in Table 1 demonstrate significant progress in sensitivity, miniaturisation, and analytical validation for a wide range of opioids and their analogues.

One of the main advantages of DBS is the minimal sample volume required, typically ranging from 2 to 50 μL. For instance, Ververi et al. [39] successfully quantified 18 drugs of abuse using only 10 μL of blood per sample, supporting the applicability of DBS in vulnerable populations and decentralised settings. Similarly, Rocca et al. [42] demonstrated that reliable analysis could be performed with as little as 2 μL, which is particularly relevant in paediatric or neonatal contexts.

The type of filter paper plays a significant role in analyte recovery and spot homogeneity. Most studies employed Whatman 903 cards, as observed in Kyriakou et al. [49], Chepyala et al. [48], and Luginbühl et al. [44] due to their widespread use and standardisation. However, alternative cards such as FTA DMPK-C [47], Capitainer^®^B [50], and QIAGEN QIAcard [13] were also explored. These studies illustrate that, while all papers support DBS application, they differ in absorption characteristics and background interference. For example, the method by Rocca et al. [42] using pre-cut cellulose filter paper yielded lower recoveries compared to the standard Whatman^®^ 903 used by Salamin et al. [45], despite offering faster drying times.

Extraction solvents and protocols varied considerably; however, methanol was the most commonly used solvent, either alone or combined with water, acetonitrile, or formic acid. For example, Shaner et al. [47] used a methanol/formic acid aqueous solution (15:85) with online solid phase extraction (SPE), while Abarca and Gerona [40] employed pure methanol with a 2-h incubation. Interestingly, even when using the same solvent, recovery rates varied. Massano et al. [14] reported low recoveries (30–50%) for a broad panel of fentanyl analogues using methanol/acetonitrile (3:1), whereas Aydoğdu et al. [16], working with traditional opioids and employing methanol with DWS buffers, achieved high recoveries for morphine (84.9–113%).

Regarding instrumentation, LC–MS/MS was the dominant analytical platform, used in over 90% of the studies. However, some authors employed advanced techniques such as ultra-high-performance liquid chromatography–high-resolution mass spectrometry–quadrupole time-of-flight (UHPLC–HRMS–QTOF) [13,14,45], supercritical fluid chromatography–tandem mass spectrometry (SFC-MS/MS) [18], or ultra-high-performance liquid chromatography-ion booster-quadrupole time-of-flight mass spectrometry (UHPLC-IB-QTOF-MS) [48]. The choice of technique significantly impacted sensitivity: Mueller et al. [18] achieved a limit of detection (LODs) of 0.5 ng/mL using SFC–MS/MS for methadone enantiomers, while Chepyala et al. [48], using UHPLC–IB–QTOF–MS, reported LODs as low as 0.2 ng/mL for fentanyl analogues. This variability highlights the potential of coupling DBS with high-resolution systems to improve detection limits.

Across the compiled data, LOD and limit of quantification (LOQ) values typically fell below 5 ng/mL, demonstrating adequate sensitivity for most forensic and clinical applications. For example, Davari et al. [32] achieved a lower limit of quantification (LLOQ) of 0.1 ng/mL for methadone and its metabolites, while Ververi et al. [50] reported LOQs of 1 ng/mL for a panel of nitazenes. Notably, some methods, such as those by Mainero et al. [41] achieved sub-ng/mL detection without prior extraction, using desorption electrospray ionisation (DESI–MS/MS).

Recovery rates varied considerably, influenced by both the chemical properties of the analytes and methodological parameters. Traditional opioids such as morphine, codeine, and methadone consistently showed high recovery rates, typically above 75% (e.g., Aydoğdu et al. [16], Kyriakou et al. [49], Chepyala et al. [38]). In contrast, synthetic opioids like brorphine or isotonitazene exhibited lower recoveries (11–27%) [50], suggesting that structural complexity and solubility must be taken into account during extraction optimisation.

Innovations in high-throughput workflows were also reported. Shaner et al. [47] implemented online SPE with flow-through desorption to streamline the analysis of fentanyl analogues, while Luginbühl et al. [44] described an automated DBS workflow for tramadol and *O*-desmethyltramadol, supporting the feasibility of DBS in routine toxicology screening.

Several studies also addressed specific challenges, including matrix effects and stereoselective analysis. Metzger et al. [43] used DBS to study methadone enantiomer distribution in mother–neonate pairs, while Vitrano et al. [13] evaluated the short-term stability of nitazenes, confirming degradation risks under certain storage conditions.

Studies such as Fariha et al. [36], introduced high-throughput or automatable formats, including electric-field-assisted elution, suggesting potential for future scalability. Others, like Zinsli et al. [35], tested DBS for drug surveillance in real-world cohorts of people who use drugs, underlining the method’s adaptability to population-level monitoring. However, despite technical consistency, some gaps remain—particularly regarding the standardisation of haematocrit correction, extraction recovery reporting, and spot homogeneity assessment.

### 4.2. Applications of DMS Using Alternative Matrices

Although DBS remains the most extensively validated DMS format, alternative matrices such as DUS, DPS, and DSS offer unique advantages in specific analytical and clinical contexts. Table 2 compiles studies employing these matrices for opioid detection, highlighting their distinct benefits, which range from non-invasiveness to extended detection windows. Their analytical performance varies considerably depending on matrix characteristics, extraction design, and detection methodology.

The sample volume used across these studies was generally consistent, typically ranging from 10 to 50 μL. Protti et al. [9] employed 20 μL of plasma to quantify oxycodone and noroxycodone with high sensitivity, whereas Ribeiro et al. [29] used only 50 μL of oral fluid on DSS cards to determine methadone and EDDP. Notably, Michely et al. [51] worked with 20 μL of urine in DUS format, covering a wide range of drug classes using comprehensive LC-MS^n^ screening. These variations suggest that the optimal sample volume is both matrix- and analyte-dependent, with smaller volumes being feasible in OF-based methods, while plasma and urine typically require higher volumes for robust quantification.

Regarding the type of paper, most methods employed cellulose-based filter cards, such as Whatman 903 or FTA DMPK variants. However, several studies, particularly those using DSS or DUS, did not specify the brand or chemical treatment of the filter paper. Ryona and Henion [54] implemented a book-type plasma spot card designed to separate plasma directly from whole blood, enabling standardised drying and high-throughput automation. Conversely, Jain et al. [52] and Almeida et al. [28] used generic filter paper for urine and OF, respectively, with limited discussion of its influence on analyte recovery or spot homogeneity. This lack of paper standardisation may partially explain some of the inter-study differences in method performance.

Extraction solvents played a critical role in analytical efficiency. For instance, Ribeiro et al. [29] used isopropanol and achieved over 50% recovery for both target analytes. Ryona and Henion [54] employed methanol-based elution with online SPE, resulting in LOQs as low as 0.25 ng/mL. In contrast, Jain et al. [52] relied on deionised water for a simple aqueous extraction prior to enzyme-linked immunosorbent assay (ELISA) analysis, which yielded poor sensitivity (LOQ > 100 ng/mL). Ribeiro et al. [29], working with DSS, observed that isopropanol was more effective than methanol for extracting methadone from dried oral fluid—a finding that underscores the importance of matrix-dependent solvent optimisation. Similarly, Michely et al. [51] used a dichloromethane: methanol (80:20) mixture post-hydrolysis in their DUS method, achieving broad-spectrum recovery across opioids and stimulants.

The predominant instrumentation was LC–MS/MS, confirming its status as the preferred platform for DMS-based analyses. This was the case in Protti et al. [9], Ribeiro et al. [29], and Ryona and Henion [54], all of whom achieved excellent sensitivity and selectivity. In contrast, Jain et al. [52] used ELISA, which resulted in markedly inferior analytical performance and lacked linear range reporting. Almeida et al. [28] and Ribeiro et al. [29] used GC–MS/MS, requiring derivatisation steps to detect volatile analytes. Although this added complexity, both methods successfully quantified opioids and metabolites in oral fluid with acceptable precision.

Recovery values varied considerably across matrices and methods. The highest recoveries (>90%) were obtained using DPS, such as in the method by Ryona and Henion [54]. DUS methods showed broader variability: Michely et al. [51] reported recoveries ranging from 56 to 95%, depending on compound class and extraction conditions, whereas Pablo et al. [53] did not report recovery values, limiting methodological evaluation. DSS methods showed more modest performance: Ribeiro et al. [29] achieved recoveries of 54.2–74.2% for methadone and EDDP. Almeida et al. [28] on the other hand, focused solely on the stability of opiates and their metabolites in DSS samples.

Sensitivity was highly dependent on both matrix and technique. In plasma, Protti et al. [9] achieved LOQs below 1 ng/mL, supporting the use of DPS in therapeutic drug monitoring. In contrast, Ryona and Henion [54] reported an LOQ of 5 ng/mL, which may be suitable for some clinical applications but reflects lower sensitivity. In urine, Michely et al. [51] achieved LODs < 1 ng/mL across multiple drug classes, including opioids and stimulants, while Jain et al. [52] reported poor sensitivity (LOQ of 100 ng/mL). DSS methods, such as that of Ribeiro et al. [29], achieved LOQs of 10 ng/mL for methadone, which, although adequate for many forensic applications, may be insufficient for low-dose clinical monitoring. Overall, plasma allowed for the highest sensitivity, followed by urine, with OF-based methods displaying greater variability.

Several studies introduced technical innovations. Ryona and Henion [54] combined a specialised DPS card with online SPE–LC–MS/MS, reducing manual handling and facilitating automation. Protti et al. [9] demonstrated the suitability of their method for clinical laboratories by eliminating derivatisation and evaporation steps, making the workflow more practical for routine use. In contrast, DSS and DUS methods remained largely manual, with fewer studies exploring automation or high-throughput formats—limiting their scalability for broader implementation.

### 4.3. Comparative Reflections Across DMS Matrices

The comparative analysis of DMS techniques across biological matrices highlights both shared benefits and matrix-specific challenges. DBS remain the most validated format, offering consistent performance in terms of sensitivity, recovery, and workflow integration. However, DPS, DUS, and DSS are gaining ground in specialised contexts due to their unique advantages.

Analytical sensitivity was highest in DBS and DPS formats, with several studies reporting LODs and LOQs below 1 ng/mL, especially when coupled with LC-MS/MS or HRMS systems [43,47,49]. DSS methods frequently showed higher LOQs (>10 ng/mL), and DUS combined with ELISA displayed limited sensitivity (LOQ ~100 ng/mL) [52], highlighting the need for matrix- and method-specific optimisation.

Recovery rates also reflected matrix compatibility. DBS and DPS consistently achieved recoveries > 85% [9,54], while DSS and DUS showed broader variability (50–80%) depending on solvent efficiency and extraction design [29]. Methanol remained the solvent of choice for blood and plasma, whereas isopropanol and dichloromethane-based mixtures were more effective in oral fluid and urine, respectively.

Several studies highlight that lower recoveries may occur for certain opioids and matrices. Classical opioids such as oxycodone and its metabolites showed satisfactory recoveries, with Protti et al. [9] reporting 80–89% in DBS, 76–85% in DUS, and 75–85% in DPS. Similarly, Aydoğdu et al. [16] observed high recovery in DUS (56–95%), supporting the robustness of urine-based spots. For DSS, Ribeiro et al. [29] reported moderate recoveries of methadone and EDDP (54–74%), reflecting matrix-specific challenges linked to oral fluid composition. In contrast, nitazene derivatives demonstrated greater variability and often poorer performance, even when assessed exclusively in DBS. Vitrano et al. [13] reported relatively high recoveries for some nitazenes (84–117%), whereas Massano et al. [14] observed more limited values (30–50%), and Ververi et al. [50] found very low recoveries (11–27%). These discrepancies highlight significant analyte-specific differences within the same format, reflecting variations in lipophilicity, analyte stability, and the strength of interactions with cellulose fibres. Support materials were predominantly cellulose-based (e.g., Whatman^®^ 903), although DPS benefitted from specialised cards allowing plasma separation and improved elution [54]. DSS and DUS studies frequently use generic paper without assessing matrix suitability, which may compromise performance.

Instrumentally, LC-MS/MS was the preferred technique across matrices, offering high sensitivity and selectivity. HRMS and UPLC platforms further enhanced detection in DBS [45], while GC-MS/MS and ELISA were occasionally applied in DSS and DUS with more limited success [29].

Scalability and automation were best demonstrated in DBS and DPS protocols, where high-throughput strategies were developed for clinical and forensic workflows [44]. DSS and DUS methods, although promising in non-invasive sampling, remain largely manual and require further optimisation and validation for routine use.

Beyond comparisons across DMS formats, several studies have directly evaluated their performance against classical sample preparation methods such as solid-phase extraction (SPE), liquid–liquid extraction (LLE), and protein precipitation (PP). Verplaetse and Henion [17] developed a fully automated DBS desorption method coupled to online SPE-LC-MS/MS for opioids, achieving LLOQs as low as 0.1–1 ng/mL with precision and linearity comparable to conventional SPE on liquid blood, while reducing preparation time and biohazard risks. Shaner et al. [47] reported similar findings for fentanyl analogues analysed in DBS with online SPE, with LODs ranging from 0.15 to 0.66 ng/mL. Protti et al. [9] compared oxycodone and its metabolites in blood and urine, analysed through both traditional and miniaturised sampling, demonstrating that DBS, DPS, and DUS achieved LOQs of 0.25–0.5 ng/mL, comparable to liquid matrix analysis (0.02–0.50 ng/mL), while offering logistical advantages. Aydoğdu et al. [16] evaluated LLE of whole blood against DBS, noting that although LLE yielded slightly higher recoveries (>90%), the variance of results obtained with the two matrices was equivalent for each analyte, with a strong correlation (r^2^ = 0.9625). Finally, Davari et al. [32] compared DBS with plasma processed by PP for methadone and its metabolites, showing that DBS recoveries (41.0–54.6%) were lower than those obtained with PP (>95%); however, DBS provided superior stability and simplified handling. Collectively, these findings confirm that DMS can deliver analytical performance comparable to classical methods, while offering clear advantages in stability, biosafety, and logistical feasibility.

**Figure 3:**
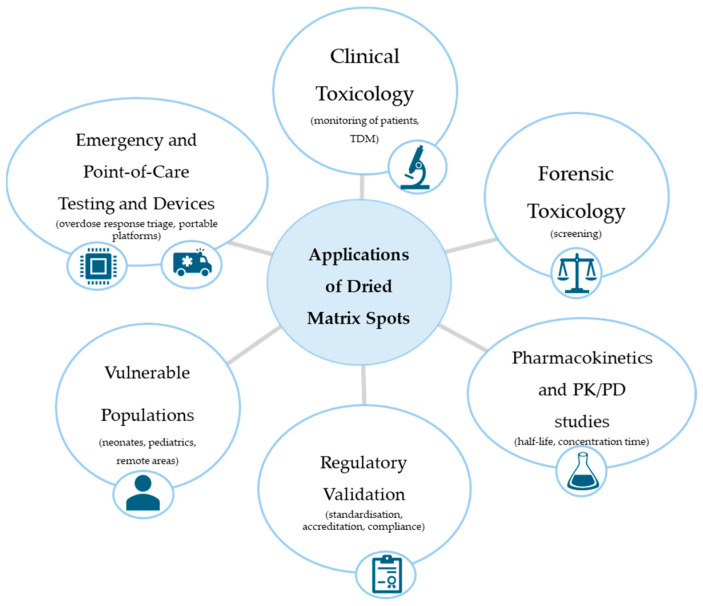
Applications of DMS techniques across diverse contexts.

In conclusion, while DBS and DPS represent robust platforms for sensitive and reproducible DMS analysis, DUS and DSS hold promise for decentralised applications if methodological improvements are implemented. Advances in paper substrates, solvent selection, and miniaturised detection systems will be key to broadening the applicability of all DMS formats in modern bioanalysis. A graphical overview of the main application domains of DMS techniques is presented in Figure 3, highlighting their growing role in clinical, forensic, and emergency settings.

## 5. Challenges, Limitations, and Future Perspectives

Despite the numerous advantages offered by DMS techniques, including portability, reduced sample volume, and logistical simplicity, several limitations remain. One of the most evident is the restricted sample volume available, which limits the possibility of reanalysis or conducting multiple tests from a single spot. This constraint becomes particularly relevant in multi-analyte panels or confirmatory workflows [7,18,20]. In addition, the complexity of biological matrices such as oral fluid or plasma introduces variable matrix effects that can impair ionisation efficiency and quantification by mass spectrometry [9,25].

Another significant barrier is the lack of standardised protocols across laboratories and studies. Variations in card type, drying time, punching strategy, and extraction solvents complicate the direct comparison of results and hinder method harmonisation [20,23,25]. Moreover, certain opioids present analyte-specific challenges, such as poor recoveries or irreversible binding to paper fibres, which necessitate the development of novel substrates or chemical derivatisation strategies [18,32].

Another aspect is the dependability of DMS results when compared to conventional liquid matrices for clinical and forensic decision-making. While several studies have found strong agreement between DMS and classical methods, particularly for opioids like methadone, oxycodone, and fentanyl analogues, and other compounds analysed in DBS or DPS with SPE- and LLE-based extractions [9,16,17,32,47]. These findings confirm that DMS can deliver sensitivity and accuracy comparable to liquid matrices, but residual concerns remain regarding matrix effects (e.g., haematocrit in DBS) and the slightly higher LOQs observed in DSS and DUS [19,21]. These factors highlight the importance of matrix-specific validation before normal deployment. In practice, real-world DMS adoption is determined not just by analytical performance, but also by logistical and economic issues. Although DMS saves costs associated with transportation, storage, and biosafety, its application in routine laboratories demands an initial investment in validation studies, workflow adaptation, and regulatory acceptance [5,7,37]. Thus, the cost–benefit ratio appears beneficial in decentralised and large-scale monitoring settings, but wider integration into clinical and forensic practice may require harmonised protocols and additional demonstrations of equivalence with liquid-based approaches.

In addition to technical and logistical issues, regulatory and accreditation requirements remain a major barrier to the wider adoption of DMS. Most accredited laboratories operate under ISO 17025 or ISO 15189 frameworks, which demand interlaboratory reproducibility and demonstrable equivalence with liquid matrices [5,7,16]. The lack of harmonised guidelines for DMS, therefore, complicates its acceptance, highlighting the need for consensus protocols and regulatory recognition before routine implementation [20,24,25].

Looking ahead, future research in the DMS field should prioritise the development of integrated systems that combine sampling, microextraction, and direct analysis using miniaturised mass spectrometry platforms [16,36,43]. In parallel, the design of novel paper substrates with tailored hydrophobic–hydrophilic properties and enhanced analyte recovery could significantly expand the applicability of DMS technologies [19,25]. The incorporation of green analytical chemistry principles, such as reduced solvent consumption, minimal waste generation, and simplified workflow, should also guide the next generation of DMS methodologies [3,19,38]. If adequately optimised and standardised, DMS-based approaches have the potential to revolutionise the monitoring of opiates and opioids, offering decentralised, reliable, and cost-effective alternatives for clinical, forensic, and epidemiological applications [5,7,16].

## 6. Conclusions

DMS-based methods represent a significant advancement in the monitoring of opiates and opioids, particularly in situations where traditional sampling is impractical or constrained by limited resources. These techniques offer several practical advantages, including low sample volume requirements, safer handling, and enhanced stability during transport and storage. Throughout this review, the diversity of DMS matrices and protocols has been emphasised, reflecting a dynamic field that continues to evolve in response to analytical, clinical, and forensic demands.

Nonetheless, several technical and operational challenges remain. These include variability in matrix effects, limited sample volume for multi-target analyses, and the absence of harmonised protocols across laboratories. Additionally, analyte-specific issues, such as low recovery or adsorption to the substrate, require further optimisation. Future efforts should focus on improving standardisation, enhancing method sensitivity, and developing integrated systems that streamline DMS workflows. With continued innovation and rigorous validation, DMS strategies are well positioned to support reliable, decentralised drug analysis in a wide range of real-world contexts.

If fully validated and implemented, DMS methodologies may play a key role in shaping the future of decentralised toxicological monitoring and personalised opioid therapy.

## Figures and Tables

**Table 1:** Analytical methods for the determination of opioids and their analogues using DBS.

Compounds	Sample Volume/Type of Paper	Sample Preparation	Instrumentation	LOD (ng/mL)	LOQ (ng/mL)	Recoveries (%)	Reference
Morphine	50 μL Whatman 903 filter paper	Dried 2 h at room temperature. Added 1 mL of solvent mixture and vortexed for 10 s. Sonicated 30 min. Centrifuged at 4100 rpm for 10 min. Dried under nitrogen at room temperature. Reconstituted with 150 μL of mobile phase and centrifuged at 14,000 rpm for 5 min.	LC–MS/MS (ESI+)	5.40	20.00	84.9–113.2	[16] (2025)
Fentanyl Morphine 6-MAM	75 µL Whatman 903 protein saver DBS cards	Added 1 mL of methanol/acetonitrile (1:1, *v*/*v*). Centrifuged at 10,000 rpm for 5 min. Evaporated using a TurboVap set to 35 °C. Reconstituted with 100 µL of 5 mM ammonium formate in deionised water (pH 3): 0.1% formic acid in methanol (90:10, *v*/*v*).	LC-MS (n.a)	n.a.	1.00	n.a	[35] (2025)
Codeine Hydrocodone Morphine Methadone Oxycodone	50 μL Whatman-903 protein saver DBS cards	Preparation DBS: Dried overnight at room temperature. Added 80 μL DWS-1 and 167 μL of DWS-2. Shaken at 45 °C for 10 min at 700 rpm. Centrifuged at 25 °C for 20 min. Preparation S-DBS: Added 80 μL DWS-1 and 167 μL of DWS-2, and vortexed for 30 s. Sonicated at 25 °C for 10 min. Preparation E-DBS: Added 80 μL DWS-1 and 167 μL of DWS-2. An electric field was applied for 3 min.	LC-MS/MS (ESI+)	n.a	1.00	n.a	[36] (2025)
Etazene Flunitazene Isotonitazene Protonitazene	30 µL QIAGEN QIAcard FTA DMPK DBS cards	Added 500 μL methanol. Sonicated 30 min, centrifuged (4000 rpm, 5 min). Dried and reconstituted in 30 μL of aqueous 0.1% formic acid: 0.1% formic acid in acetonitrile (80:20, *v*/*v*). Vortexed and centrifuged.	UHPLC-HRMS/MS (HESI)	0.25–0.35	0.50	84.00–117.00	[13] (2024)
Remifentanil	20 µL Whatman 903 DBS cards	Dried for 2 h at room temperature. Extracted with 0.5 mL of methanol/water (1:1, *v*/*v*) with 1% formic acid (*v*/*v*). SPE: elution: 1 mL methanol.	LC-MS/MS (ESI+)	n.a	0.30	82.00–83.00	[37] (2024)
Tramadol Morphine Hydromorphone Methadone Oxycodone 6-MAM Codeine Buprenorphine	10 µL Capitainer qDBS cards	Dried. Added 10 µL of pure water, waited 5 min, added 300 µL of acetonitrile and 3.3% ethylene glycol. Shaken for 15 min and centrifugation (2000× *g*) for 5 min. Added 300 µL of methanol and acetonitrile with 1% formic acid for extraction (1:1, *v*/*v*). Shaken for 15 min and centrifugation (2000× *g*) for 5 min. Dried and reconstituted in 70 µL of 25% methanol.	LC-MS/MS (n.a)	0.10–0.30	n.a	n.a	[38] (2024)
6-MAM Buprenorphine Codeine EDDP Methadone Morphine Norbuprenorphine	10 μL Capitainer^®^B cards	Dried ≥ 3 h at room temperature in the dark. Added 500 μL methanol. Stirred and sonicated for 30 min at room temperature. Dried under nitrogen. Reconstituted with 30 μL of 5 mM formic acid in water: 5 mM formic acid in acetonitrile (1:1, *v*/*v*). Centrifuged for 5 min at 4000 rpm.	UHPLC-MS/MS (ESI+)	0.50–1.00	1.00	34.00–103.00	[39] (2024)
Hydrocodone	30 μL Thick cellulose cardstock	Dried overnight. Added 200 μL of methanol. Incubated for 2 h at 37 °C. Reconstituted in 150 μL of a water: acetonitrile (90:10, *v*/*v*). Centrifuged for 10 min at 14,000 rpm.	HPLC-MS/MS (ESI+)	0.05 ng/mL	LLOQ: 0.10 ng/mL	2.00%	[40] (2023)
4-Fluorobutyrfentanyl 4-Methylfentanyl Acetylfentanil AH-7921 Alfentanil Acrylfentanyl β-Phenylfentanyl Butyrfentanyl Butyrylfentanyl (carboxy metabolite) Butyrylnorfentanyl Carfentanil Cyclopropylfentanyl Despropionyl p-Fluorofentanyl Fentanyl Furanylfentanil Furanylnorfentanyl Hydrocodone Hydroxyfentanyl Hydroxythiofentanyl MT-45 Norfentanyl OcFentanyl Phenylacetylfentanil Remifentanil Sufentanil Tramadol U-47700 Valerylfentanyl (carboxy metabolite)	30 μL FTA™ DMPK C cards	Dried ≥ 3 h. Added 500 μL methanol/acetonitrile (3:1, *v*/*v*). Ultrasonication for 30 min at room temperature. Centrifuged for 5 min at 13,000× *g* and left to dry. Reconstituted with 50 μL methanol, centrifuged for 5 min at 13,000× *g*.	UHPLC-MS/MS-QTOF (ESI+)	2.00–5.00	5.00	30.00–50.00	[14] (2022)
Fentanyl Tramadol	2 μL pre-cut cellulose filter paper	Dried. Added 50 μL of water and 50 μL of acetonitrile shaking (5 min) and centrifugation (8 min, 6797 rpm, 20 °C)	DESI-MS/MS UHPLC-MS/MS (n.a)	50.00–80.00 pg/mm^2^	150.00–240.00 pg/mm^2^	n.a	[41] (2022)
Tramadol Fentanyl	2 µL pre-cut cellulose filter paper	Dried for 10 min. Added 50 µL of distilled water and sonicated for 5 min. Added 50 µL of acetonitrile, sonicated for 5 min. Centrifuged for 8 min at 8000 rpm and 20 °C.	UPLC-MS/MS (ESI+)	0.10–0.50	0.30–1.50	89.00–97.00	[42] (2021)
*R*-methadone *S*-methadone *R*-/*S*-EDDP	10 µL Whatman 903 Paper Saver Snap Apart cards	Dried for at least 3 h at room temperature. Added 100 µL methanol, vortexed for 30 s and incubated for 30 min.	LC-MS/MS SFC-MS/MS	LC-MS/MS 1.50–2.50 SFC-MS/MS 0.50	LC-MS/MS 5.00–10.00 SFC-MS/MS 1.00–20.00	85.00–100.00	[18] (2021)
*S*-Methadone *R*-Methadone *S*-EDDP *R*-EDDP	n.a Whatman DMPK-90 cards	Microcentrifuged. Extracted with ethyl acetate. Dried, and reconstituted with 50 μL of 10% acetonitrile in 0.1% formic acid (pH 6.5).	HPLC MS/MS (n.a)	n.a	0.50–1.00	n.a	[43] (2021)
Methadone EDDP EMDP	50 μL Whatman 903 filter paper cards	Added 420 µL of methanol/0.2 M of zinc sulfate (7:3, *v*/*v*). Vortexed for 2.5 min. Centrifuged at 13,000× *g* at 4 °C for 8 min.	LC-MS/MS (ESI+)	n.a	LLOQ: 0.10	41.00–54.60	[32] (2020)
Tramadol *O*-desmethyltramadol	240 μL BioSample TFN filter paper, AutoCollect™ DBS cards	Dried ≥ 3 h at 21 °C. Extracted with water: methanol mixture (90/10, *v*/*v*).	LC-MS/MS (ESI+)	2.00–5.00	20.00–25.00	16.00–62.00	[44] (2020)
Tramadol *O*-desmethyltramadol *N*-desmethyltramadol	10 μL Whatman^®^ protein saver card 903	Dried ≥ 1 h at room temperature. Added 100 μL of methanol. Incubated for 15 min (vortex for 5 s every 5 min). Diluted 50 μL supernatant with 50 μL water.	UHPLC-HRMS (ESI+) UPLC-MS/MS (ESI+)	5.00	n.a	48.90–68.90	[45] (2020)
3-Methylfentanyl Alfentanil α-Methylfentanyl Carfentanil Fentanyl Lofentanyl Sufentanil Norcarfentanil Norfentanyl Norlofentanyl Norsufentanil Cyclopropylfentanyl 2-Furanylfentanyl Acryloyfentanyl Isobutyrylfentanyl Octylfentanyl Methoxyacetylfentanyl	5 μL Whatman 903 protein saver cards	Added 1.0 mL of methanol/acetonitrile (1:1, *v*/*v*). Mixed at 1000 rpm for 10 min. Dried under a stream of 60 °C nitrogen. Reconstitution with 100 μL of a water: acetonitrile solution containing 0.1% formic acid (90:10, *v*/*v*). Mixed at 1000 rpm for 5 min	LC-MS/MS (ESI+)	0.10–0.30	1.00	63.00–91.00	[46] (2019)
Oxycodone Noroxycodone Oxymorphone	n.a. Whatman 903 card	Dried 1.5 min (MAD, 700 W). Added 500 μL methanol. Sonicated 1 min, centrifuged (1400× *g*, 1 min). Dried and reconstituted in 50 μL of 0.1% formic acid in acetonitrile and 0.1% formic acid in water (5:95, *v*/*v)*	LC-MS/MS (ESI+)	0.15	0.50	80.00–89.00	[9] (2018)
Fentanyl Sufentanil Carfentanil Alfentanil Lofentanyl α-Methylfentanyl 3-Methylfentanyl	5 μL FTA DMPK-C blood spot cards	Dried for ≥2 h. Desorbed with 1.2 mL methanol/aqueous 1% formic acid (15:85, *v*/*v*) at 100 °C. Trapped on SPE cartridge (preconditioned).	HPLC-MS/MS (ESI+)	0.15–0.66	n.a	n.a	[47] (2017)
Morphine Codeine Methadone Fentanyl Nalorphine Dihydrocodeine Tramadol Butorphanol	25 μL Whatman 903 card	Dried for 2 h at room temperature. Added 200 μL of 80% acetonitrile for 5 min. Centrifuged at 15,000× *g* for 5 min. 170 μL of the supernatant was dried, reconstituted with 150 μL of deionised water.	UHPLC-IB-QTOF-MS (ESI±)	0.20–2.90	n.a	71.38–113.13	[48] (2017)
Morphine Codeine 6-MAM Methadone EDDP	30 μL Whatman 903 Protein Saver cards	Added 990 μL methyl alcohol. Sonicated for 15 min Centrifuged at 3500× *g* for 5 min. Dried under vacuum. Reconstituted with 100 μL mobile phase.	UHPLC-MS/MS (ESI+)	1.50	5.00	79.50–89.20	[49] (2016)

AH-79213—4-dichloro-*N*-[(1-dimethylamino)cyclohexylmethyl]benzamide; 6-MAM—6-Monoacetylmorphine; DESI-MS/MS—Desorption Electrospray Ionisation–Tandem Mass Spectrometry; DWS-1—Daily Working Solution-1; DWS-2—Daily Working Solution-2E-DBS—Electromembrane-Assisted Dried Blood Spot; EDDP—2-Ethylidene-1,5-dimethyl-3,3-diphenylpyrrolidine (methadone metabolite); ESI+—Electrospray Ionisation in Positive Mode; HESI—Heated Electrospray Ionisation; HPLC—High-Performance Liquid Chromatography; HRMS—High-Resolution Mass Spectrometry; IB-QTOF—Ion Booster Quadrupole Time-of-Flight; LC-MS—Liquid chromatography–mass spectrometry; LC-MS/MS—Liquid Chromatography–Tandem Mass Spectrometry; LLOQ—Lower Limit of Quantification; LOD—Limit of Detection; LOQ—Limit of Quantification; MT-45—1-cyclohexyl-4-(1,2-diphenylethyl)piperazine; n.a.—Not Applicable; QTOF—Quadrupole Time-of-Flight; S-DBS—Sonic-Assisted Dried Blood Spot; SFC-MS/MS—Supercritical Fluid Chromatography–Tandem Mass Spectrometry; SPE—Solid-Phase Extraction; UHPLC-MS/MS—Ultra-High-Performance Liquid Chromatography–Tandem Mass Spectrometry; U-47700—3,4-dichloro-*N*-[2-(dimethylamino)cyclohexyl]-*N*-methylbenzamide.

**Table 2:** Analytical methods for the determination of opioids and their analogues using alternative DMS (The studies are organised according to the biological matrix analysed).

Compounds	Matrix	Sample Volume/Type of Paper	Sample Preparation	Instrumentation	LOD (ng/mL)	LOQ (ng/mL)	Recoveries	Reference
*R*-methadone *S*-methadone *R*-/*S*-EDDP	Humour vitreous	10 µL Whatman 903 Paper Saver Snap Apart cards	Dried for ≥3 h at room temperature. Added 100 µL methanol. Vortexed for 30 s and incubated for 30 min.	LC-MS/MS SFC-MS/MS	LC-MS/MS 1.50–2.50 SFC-MS/MS 0.50	LC-MS/MS 5.00–10.00 SFC-MS/MS 1.00–20.00	85.00–100.00	[18] (2021)
*R*-methadone *S*-methadone *R*-/*S*-EDDP	Pericardial fluid	10 µL Whatman 903 Paper Saver Snap Apart cards	Dried for ≥3 h at room temperature. Added 100 µL methanol. Vortexed for 30 s and incubated for 30 min.	LC-MS/MS SFC-MS/MS	LC-MS/MS 1.50–2.50 SFC-MS/MS 0.50	LC-MS/MS 5.00–10.00 SFC-MS/MS 1.00–20.00	85.00–100.00	[18] (2021)
Morphine Codeine 6-MAM	Oral Fluid	50 µL Whatman™ 903 protein saver card	Samples were dried for 12 h, after which 3 mL of methanol was added. They were mixed for 5 min on a roller at room temperature and then centrifuged for 15 min at 3500 rpm. The supernatant was dried under nitrogen and derivatised with 50 µL of MSTFA containing 5% TMCS for 2 min in a microwave oven at 800 W.	GC-MS/MS (EI+)	n.a	n.a	n.a	[28] (2022)
Methadone EDDP	Oral Fluid	50 µL Whatman™ 903 protein saver cards	Dried overnight. Added 1 mL of isopropanol. Mixed 1 min at 70 rpm on a roller-mixer shaker. Centrifuged at 3500 rpm at 4 °C for 15 min. Dried under nitrogen. Reconstituted in 50 μL of methanol.	GC-MS/MS (EI+)	5.00	10.00	54.20–74.20	[29] (2019)
Oxycodone Noroxycodone Oxymorphone	Urine	20 µL Whatman FTA™ DMPK-B IND card	Samples were subjected to MAD treatment at 700 W for 1.5 min, followed by the addition of 500 μL of methanol. UAE was performed for 1 min, and the samples were then centrifuged at 1400× *g* for 1 min. The supernatant was dried and reconstituted in 50 μL of 0.1% formic acid in acetonitrile and 0.1% formic acid in water (5:95, *v*/*v*)	LC-MS/MS (ESI+)	0.20	0.50	76.00–85.00	[9] (2018)
Codeine Morphine Buprenorphine	Urine	20 µL Whatman 903 protein saver cards	Dried overnight. Added 500 μL of a glucuronidase/arylsulfatase solution in 100 mM aqueous ammonium acetate (1:20, *v*/*v*). Extracted with dichloromethane-methanol (1:1) containing 3% 1 M aqueous ammonium carbonate at pH 9. Evaporated to dryness under a nitrogen stream at 70 °C and reconstituted in 100 µL methanol.	LC-MS^n^ (ESI)	200.00 (LOI)	n.a	56.00–95.00	[51] (2017)
Morphine	Urine	20 µL Whatman Filter paper 903	Dried overnight. Added 500 µL of deionised water. Incubated 24 h at 37 °C (water bath shaker).	ELISA	100.00	n.a	99.40%	[52] (2019)
6-MAM Buprenorphine Tramadol Codeine EDDP Fentanyl Hydrocodone Hydromorphone Methadone Morphine Norbuprenorphine Fentanyl Norfentanyl Noroxycodone Oxycodone Tapentadol	Urine	10 µL PerkinElmer 226 Bioanalysis RUOCards	Dried before analysis.	LC-HRMS/MS (HESI+)	10.00–100.00	n.a	n.a	[53] (2020)
Oxycodone Noroxycodone Oxymorphone	Plasma	10 µL Whatman FTA™ DMPK-B IND card	Samples were subjected to MAD treatment at 700 W for 1.5 min, followed by the addition of 500 μL of methanol. UAE was performed for 1 min, after which the samples were centrifuged at 1400× *g* for 1 min. The supernatant was dried and reconstituted in 50 μL of 0.1% formic acid in acetonitrile and 0.1% formic acid in water (5:95, *v*/*v*).	LC–MS/MS (ESI+)	0.08	0.25	75.00–85.00	[9] (2018)
Morphine Codeine Oxycodone Hydrocodone	Plasma	50 µL Perkin Elmer 226 cards	All 500 μL of blood contained disodium ethylenediaminetetraacetate. Incubated at 37 °C with 200 rpm agitation for 30 min. Rested 30 min before DPS preparation.	LC-MS/MS (ESI+)	n.a	5.00 ng/mL	93.20–97.80	[54] (2016)

6-MAM—6-Monoacetylmorphine; DESI—Desorption Electrospray Ionisation; DPS—Dried plasma spot; EDDP—2-Ethylidene-1,5-dimethyl-3,3-diphenylpyrrolidine (methadone metabolite); EI+—Electron ionisation in positive mode; ELISA—Enzyme-Linked Immunosorbent Assay; ESI+—Electrospray Ionisation in Positive Mode; GC-MS/MS—Gas Chromatography–Tandem Mass Spectrometry; HESI+—Heated Electrospray Ionisation in Positive Mode; LC-HRMS/MS—Liquid chromatography–high-resolution tandem mass spectrometry; LC-MS/MS—Liquid Chromatography–Tandem Mass Spectrometry; LC-MS^n^—Liquid Chromatography with Multistage Tandem Mass Spectrometry; MSTFA—*N*-Methyl-*N*-(trimethylsilyl)trifluoroacetamide; LOD—Limit of Detection; LOI—Limit of Identification; LOQ—Limit of Quantification; MAD—Microwave-Assisted Drying; n.a.—Not Applicable; SFC—Supercritical Fluid Chromatography; TMCS—Trimethylchlorosilane; UAE—Ultrasound-Assisted Extraction.
